# Stereological Assessments of Neuronal Pathology in Auditory Cortex in Schizophrenia

**DOI:** 10.3389/fnana.2017.00131

**Published:** 2018-01-09

**Authors:** Emily M. Parker, Robert A. Sweet

**Affiliations:** ^1^Department of Psychiatry, University of Pittsburgh, Pittsburgh, PA, United States; ^2^Department of Neurology, University of Pittsburgh, Pittsburgh, PA, United States; ^3^VISN 4 Mental Illness Research, Education and Clinical Center (MIRECC), VA Pittsburgh Healthcare System, Pittsburgh, PA, United States

**Keywords:** auditory cortex, neurostereology, schizophrenia, postmortem, dendritic spine

## Abstract

It has long been known that auditory processing is disrupted in schizophrenia. More recently, postmortem studies have provided direct evidence that morphological alterations to neurons in auditory cortex are implicated in the pathophysiology of this illness, confirming previous predictions. Potential neural substrates for auditory impairment and gray matter loss in auditory cortex in schizophrenia have been identified, described, and are the focus of this review article. Pyramidal cell somal volume is reduced in auditory cortex, as are dendritic spine density and number in schizophrenia. Pyramidal cells are not lost in this region in schizophrenia, indicating that dendritic spine reductions reflect fewer spines per pyramidal cell, consistent with the reduced neuropil hypothesis of schizophrenia. Stereological methods have aided in the proper collection, reporting and interpretation of this data. Mechanistic studies exploring relationships between genetic risk for schizophrenia and altered dendrite morphology represent an important avenue for future research in order to further elucidate cellular pathology in auditory cortex in schizophrenia.

## Introduction

Schizophrenia is a chronic illness that besets approximately 1% of the global population (Wong and Van Tol, 2003) and auditory impairment is a fundamental characteristic of schizophrenia psychopathology. Most commonly, individuals with schizophrenia present with auditory hallucinations (Insel, 2010). Further, many individuals with this illness experience auditory sensory processing deficits that can manifest, for instance, in impaired ability to distinguish between auditory tones (Pekkonen et al., 2002; O’Donnell et al., 2004; Kantrowitz et al., 2011, 2014). Auditory sensory processing deficits in turn contribute to socio-cognitive dysfunction (Leitman et al., 2005, 2007, 2010; Javitt and Sweet, 2015; Kantrowitz et al., 2015, 2016). Unlike the positive symptoms of schizophrenia, socio-cognitive dysfunction is not targeted by available pharmacological interventions. Among individuals with schizophrenia, those with prominent socio-cognitive dysfunction have the poorest functional outcomes (Green et al., 2004, 2015; Fett et al., 2011).

Auditory sensory processing deficits typically emerge in schizophrenia around the time of the first psychotic episode and persist over the course of the illness (McCarley et al., 1991; Holcomb et al., 1995; Strous et al., 1995; Javitt et al., 1997; Wexler et al., 1998; Leitman et al., 2005, 2010; Kantrowitz et al., 2011; Gold et al., 2012; Jahshan et al., 2013). Electroencephalography studies reveal that individuals with schizophrenia exhibit reduced auditory mismatch negativity (MMN) responses (Shelley et al., 1991; Javitt, 1993; Catts et al., 1995; Michie et al., 2000; Kasai et al., 2002). MMN is an event-related potential recorded immediately following a stimulus that differs in characteristic from preceding stimuli (for example a tone of a deviant pitch among a series of tones of the same pitch) and, in the auditory system, reflects pre-attentive auditory sensory processes. In schizophrenia, reduced auditory MMN is correlated with impaired auditory tone discrimination (Javitt et al., 1994, 1996, 2000; Leitman et al., 2010). Electroencephalography methods likewise indicate that individuals with schizophrenia exhibit impaired auditory steady-state response (aSSR) entrainment, predominantly in the gamma frequency range (Brenner et al., 2009; Hamm et al., 2011, 2015). Altered fast GABAergic inhibition in auditory circuits is presumed to underlie impaired aSSR entrainment in this illness (Kwon et al., 1999; Light et al., 2006; Krishnan et al., 2009).

Cortical gray matter loss is a hallmark anatomical feature of schizophrenia (Zipursky et al., 1992; Schlaepfer et al., 1994; see this review, Shenton et al., 2001). The most pronounced gray matter loss is observed in frontal and temporal regions (Wong and Van Tol, 2003), most notably in the superior temporal gyrus (STG; McCarley et al., 1999; Shenton et al., 2001; Honea et al., 2005). Gray matter volume reduction in the STG ranks among the most consistent findings from studies reporting gray matter loss in schizophrenia (McCarley et al., 1999). Gray matter loss is apparent around the time of schizophrenia onset (Hirayasu et al., 1998, 2000; Kubicki et al., 2002; Kasai et al., 2003) and in the years following first episode psychosis (Menon et al., 1995; van Haren et al., 2007; see this review, Vita et al., 2012). Gray matter reductions occur within the STG in Heschl’s gyrus (Hirayasu et al., 2000) and the planum temporale (Barta et al., 1997; Kwon et al., 1999; Hirayasu et al., 2000), where the primary auditory cortex (A1) and auditory association cortex are located, respectively.

Functional MRI studies reveal that auditory MMN reductions in schizophrenia subjects are correlated with gray matter loss in Heschl’s gyrus (Salisbury et al., 2007). Similarly, auditory MMN and tone discrimination are thought to depend on the integrity of cells in supragranular layers (I–III) of the A1 (Javitt et al., 1994). Thus, we have hypothesized that neurons within the supragranular layers of A1 are altered in schizophrenia (Lewis and Sweet, 2009; Javitt and Sweet, 2015). Electroencephalography and *in vivo* imaging approaches do not have the resolution to test this prediction directly, requiring human postmortem studies to assess gray matter alterations at the neuronal level. In theory, a wide array of neuronal aberrations could lead to reduced gray matter volumes in schizophrenia, as gray matter has multiple constituents, including, neurons, glia and endothelial cells, the cell bodies of these cells and their neuropil, which is made up of the unmyelinated portions of axons, dendrites and the processes of glia. Therefore, it is conceivable that auditory cortical neurons could be altered in schizophrenia due to: (1) reduced neuron somal size; (2) reduced number of axon boutons; (3) reduced number of dendritic spines; (4) fewer total cells; or (5) a combination of any two or more of these possibilities.

The following review is a synopsis of human postmortem studies conducted by our lab that reveal neuronal abnormalities in auditory cortex in schizophrenia. These studies provide direct evidence that morphological features of neurons in the human auditory cortex are implicated in the pathology of this illness. Likewise, these findings provide potential neural correlates for auditory sensory processing deficits and cortical gray matter loss in primary and secondary auditory regions in schizophrenia. Descriptions of the brain regions, predominant feedforward circuit and principal cells in the ascending auditory pathway are provided as background. Next, morphometric findings revealing neuronal pathology in auditory cortex in schizophrenia are detailed. Finally, stereological methods are emphasized in this review that: (1) aided in the collection and interpretation of schizophrenia auditory cortex data; (2) advanced the field at each step; and (3) significantly strengthened the validity of the published results.

## Anatomical Constituents, Circuits and Cells of the Ascending Auditory Pathway

The STG is located in the bilateral temporal lobe, just below the Sylvian fissure. The posterior half of the STG in human is often further categorized into two sub-regions, Heschl’s gyrus, and the region posterolateral to Heschl’s gyrus, the planum temporale. These sub-regions contain the human auditory cortex, which is comprised of the A1 and auditory association cortex (A2). A1 is located in Heschl’s gyrus and was historically referred to as Brodmann area 41 (BA41). A2 is located posterolateral to A1 in the STG in an area that spans portions of both Heschl’s gyrus and the planum temporale. A2 is also known as Brodmann area 42 (BA42; Garey, 1909/1994; Rivier and Clarke, 1997; Nakahara et al., 2000; Hackett et al., 2001; Wallace et al., 2002; Sweet et al., 2005).

**Figure 1:**
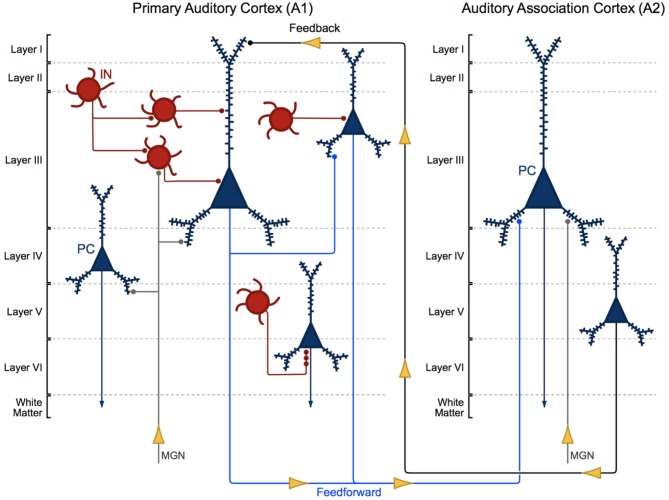
Cytoarchitecture of feedforward and feedback auditory circuits. Thalamic projections from the medial geniculate nucleus (MGN) synapse onto pyramidal cells (PCs, blue) and interneurons (INs, red). Layer III PCs in primary auditory cortex (A1) send local intralaminar projections to other layer III PCs in this region and longer-range feedforward projections to PCs in layer III of auditory association cortex (A2). Layer V PCs in A2 in turn send excitatory feedback projections to neurons in layer I in A1.

Auditory information travels in a feedforward circuit through several structures in the ascending auditory pathway before reaching the auditory cortex in the STG. Human and non-human primate auditory regions exhibit significant cytoarchitectural and functional homology (Hackett et al., 2001; Dick et al., 2012), thus, findings from non-human primate studies have been used to infer the functional organization of the human ascending auditory pathway. Briefly, auditory information travels from hair cells in the inner ear to the cochlear nucleus, superior olive, inferior colliculus and then to the medial geniculate nucleus (MGN) in the thalamus (Hackett, 2011). MGN thalamic axons synapse onto the dendrites of pyramidal cells and interneurons located in deep layer III and layer IV (Pandya and Rosene, 1993; Hashikawa et al., 1995; Molinari et al., 1995; Pandya, 1995), the major recipient layers of feedforward projections in A1 (Hackett, 2011). From there, intralaminar projections from deep layer III pyramidal cells activate A1 pyramidal cells in layer III (Mitani et al., 1985; Ojima et al., 1991; Wallace et al., 1991), to complete what is generally known as the ascending auditory pathway (Figure 1).

Layer III A1 pyramidal cells project to pyramidal cells in other layers within A1 and to pyramidal cells in layer III of A2. Layer III and V pyramidal cells in A2 send feedback projections to layer I in A1 (Pandya and Sanides, 1973; Galaburda and Pandya, 1983; Pandya and Rosene, 1993; Figure 1). The molecular and morphological properties and axonal targeting patterns of cortical GABAergic inhibitory interneurons are diverse. Calbindin and somatostatin expressing interneurons provide inhibitory input to the distal dendrites of pyramidal cells. Calretinin and vasoactive intestinal polypeptide expressing interneurons modulate the activity of other cortical interneurons. Parvalbumin expressing basket cell interneurons provide inhibitory input to the somas and proximal dendrites of nearby pyramidal cells (Kepecs and Fishell, 2014). Finally, parvalbumin expressing chandelier cells provide GABAergic inhibitory input to the axon initial segment of pyramidal cells (Somogyi, 1977; Fairén and Valverde, 1980; DeFelipe et al., 1985; Figure 1; A1, bottom right).

Pyramidal cells are the principal cells of the auditory cortex and are recognized by their characteristic pyramid-shaped soma, distinctive radially oriented apical dendrite and multiple basilar dendrites. The dendrites of pyramidal cells have small protrusions, known as dendritic spines, which are the major recipient sites for excitatory synaptic transmission in the brain (Gray, 1959). Excitatory inputs to pyramidal cells almost exclusively synapse onto dendritic spines (Spacek and Harris, 1998; Arellano et al., 2007; Chen et al., 2012), interneurons primarily synapse onto the dendritic shaft of pyramidal cells and less frequently synapse onto spines (Somogyi and Cowey, 1981; DeFelipe et al., 1989; Yuste, 2011; Chen et al., 2012). Spines are rapidly motile structures (Fischer et al., 1998; Dunaevsky et al., 1999) that often undergo morphological alterations in an activity-dependent manner. Dendritic spine form and function are inextricably linked, and altered spine morphology impacts both synaptic transmission and plasticity. The role of dendritic spines in shaping neuronal system function has been extensively debated (Yuste, 2011). One view holds that spiny synapses are altered, and the morphology and molecular profile of spines are tuned, to regulate the gain of pyramidal cell input/output properties toward the reorganization of neural networks.

### Studies of Pyramidal Cell Somal Volume (Sweet et al., 2003, 2004)

#### Stereologic Methods

Initial studies of the morphometric properties of auditory cortex neurons (Sweet et al., 2003, 2004) did not fully implement uniform random sampling, but did make use of components of a stereologic sampling scheme to select brain tissue from schizophrenia and unaffected comparison cases. Brain specimens were acquired at the time of autopsy and assigned consensus diagnoses by a panel of experienced clinicians using DSM-IV criteria as described previously (Glantz and Lewis, 2000). Following brain extraction, the left temporal lobe was cut into 1–2 cm thick coronal slabs. From these slabs, a single slab containing STG with a visible Heschl’s gyrus and planum temporale was selected, a non-uniform random approach, for each case. STG slabs were cut into 40 μm thick sections on a cryostat. Next, tissue sections were selected using a systematic uniformly random sampling (SURS) approach, and stained for Nissl substance. The Nissl-stained sections were examined, and a subset of three Nissl-stained sections were selected using a set of pre-defined criteria (non-uniform random). These criteria required that: (1) each section contain A1 and A2 oriented perpendicular to the pial surface to allow clear visualization of cortical layers and identification of pyramidal cells; and (2) each successive section selected must be no less than 1600 μm away from any other selected section (Sweet et al., 2003).

Pyramidal cells within the deepest 1/3 of layer III of auditory cortex were the focus of these studies. Pyramidal cells in layer V were also assessed. The deepest 1/3 of layer III or layer V was delineated for each section, and pyramidal cells within these regions were sampled systematic uniformly random (within sections) using the optical dissector (Gundersen, 1986), as implemented in Stereo Investigator software (Microbrightfield Inc., Colchester, VT, USA). Images of cell bodies were captured using a 1.4 numerical aperture, 100× oil immersion objective. Somal volumes of pyramidal cells were then estimated using the nucleator (Gundersen, 1988), with one exception, as implemented within Stereo Investigator software. The nucleator is a stereological method that uses isotropic test lines to estimate number-weighted mean particle (in this case somal) volume. This approach requires isotropic tissue sections, which have been, by definition, systematically and randomly sampled in three planes (x, y, z; Gundersen et al., 1988). As is evident from the above description of tissue preparation, the use of the nucleator employed by the authors did not ensure the requirement for isotropic orientation of the sampled neurons. Statistical tests were performed on pairs of subject cases in these studies as well as in each study described in this review article. In this study and in each of the studies detailed in this review, specimens were assessed in pairs, comprising tissue from each diagnostic group. Thus, each pair consisted of one schizophrenia case and one unaffected comparison case matched for age, sex, postmortem interval and, to the extent possible, handedness.

#### Results

**Figure 2:**
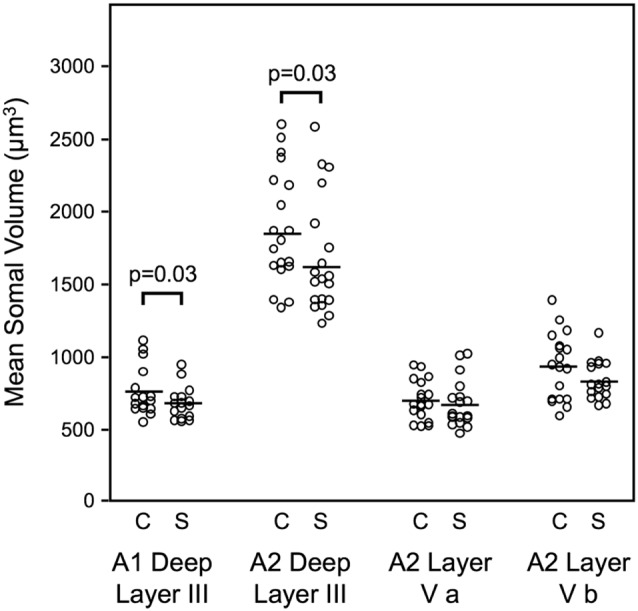
Reduced pyramidal cell somal volume in schizophrenia (Sweet et al., 2004). Summary of findings of mean pyramidal cell somal volume in schizophrenia including in deep layer III of A1, deep layer III, layer V a and layer V b of A2. Studies of auditory cortical pyramidal cell somal volume (Sweet et al., 2003, 2004) revealed significantly reduced pyramidal cell somal volume in deep layer III of A1, and in deep layer III of A2 in schizophrenia.

Mean somal volume of deep layer III pyramidal cells was reduced in schizophrenia by 13.1% in auditory association cortex (Sweet et al., 2003). Similarly, mean pyramidal cell somal volume was decreased in schizophrenia by 10.4% in deep layer III of A1, with no change in the somal volume of layer V pyramidal cells in A2 (Sweet et al., 2004; Figure 2). These data implicated feedforward but not feedback auditory circuits in schizophrenia and provided evidence for neuronal morphological abnormalities consistent with gray matter loss in auditory cortex in schizophrenia. Importantly, these studies provided the first direct demonstration of human auditory cortex pathology in schizophrenia.

### Studies of Axon Boutons

#### Study of Synaptophysin-Immunoreactive Boutons (Sweet et al., 2007)

##### Stereologic methods

Brain specimens were acquired and sampled using the approaches used for studies of pyramidal cell somal volume detailed above. The sections adjacent to those used in the prior studies were selected and processed for immunolabeling with an antibody for synaptophysin. Synaptophysin is a presynaptically located synaptic vesicle glycoprotein that labels approximately 95% of cortical axon boutons (Jahn et al., 1985; Navone et al., 1986). Synaptophysin immunoreactivity (SYP-IR) was visualized with a diaminobenzidine reaction product followed by osmium tetroxide stabilization. Layer I of A1 and deep layer III of A1 and A2 were delineated in adjacent sections stained for Nissl substance and the outlined regions were then aligned with the SYP-IR sections using surface fiduciaries. Because the sampling of auditory cortex deviated from uniform random, it was not possible to estimate the volume of the regions of interest or determine SYP-IR object number. Instead, only a biased estimate of SYP-IR object density was determined.

Poor antibody penetration and the potential for loss of structures at the cut surface are two issues that should be addressed when using thick tissue sections for immunohistochemistry. Thus, pilot studies using the optical dissector (Gundersen, 1986) to sample SYP-IR objects in z-axis depths of 0.5 μm intervals were conducted to assess antibody penetration and to sample cases in a zone of z-axis depth with uniformly high penetration across cases, and free from artifactual loss of labeled boutons (Sweet et al., 2007).

##### Results

Putative bouton density (SYP-IR object density) was decreased 13.6% in deep layer III in A1 in schizophrenia, relative to unaffected comparison cases. In contrast, SYP-IR density in layer III of A2 and in layer I of A1 were both not significantly altered in schizophrenia. Putative bouton density reductions did not correlate with reduced somal volume in either region (Sweet et al., 2007), suggesting that the input/output properties of pyramidal cells in auditory feedforward and feedback circuits exhibit more nuanced properties than originally expected, although, this may only be true in the context of pathology.

It must be pointed out that the above study was limited by the biased nature of the sampling. Neither A1 nor A2 reference volume could be estimated using the approaches employed. Without total reference volume, density estimations suffer from the reference trap (Braendgaard and Gundersen, 1986). Thus, the finding of reduced axon bouton density in A1 deep layer III could reflect either reduced number of SYP-IR structures or expanded tissue volume of the sampled region with no change in number of SYP-IR structures. The former interpretation was viewed as more likely given the evidence of auditory cortical gray matter reduction in schizophrenia (Shenton et al., 2001), but could not be established by the current study. For the same reason, it is also conceivable that unchanged SYP-IR object densities in layer I of A1 and in layer III of A2 could reflect modestly lower numbers of SYP-IR structures in schizophrenia.

#### Study of Vesicular Glutamate Transporter-Immunoreactive Boutons (Moyer et al., 2013)

##### Stereologic methods

Brain specimens were collected and assigned consensus diagnoses as described in previous studies (Sweet et al., 2003, 2004, 2007). Two cohorts, each with one schizophrenia group and one unaffected comparison group, were assessed. Cohorts 1 and 2 are distinguished by the cutting and sampling methods employed. The subjects included in Cohort 1 were the same as in “Study of Synaptophysin-Immunoreactive Boutons (Sweet et al., 2007)” section above, and the tissue sections for this cohort were cut and sampled using the procedures detailed above (Sweet et al., 2003, 2004, 2007). In contrast, new cases in Cohort 2, comprising the two diagnostic groups, were processed using a rigorous, unbiased SURS method (Dorph-Petersen et al., 2009). The entire left STG was dissected out of coronal blocks of brain specimens for the generation of Cohort 2 tissue. Pial surfaces of STG blocks were then painted with hematoxylin and reassembled in 7% low-melt agarose based on original *in vivo* orientation. Next, STG blocks were cut into 3 mm slabs orientated orthogonal to Heschl’s gyrus. From these SURS slabs, every other slab with a random start was selected for A1 mapping and processed using a Nissl protocol to reveal the cytoarchitectural features of the tissue. The sections adjacent to each Nissl section were processed with AChE or parvalbumin to reveal chemoarchitectural characteristics of the tissue. Cyto- and chemoarchitectural sections were used to identify the boundaries of the primary and secondary auditory cortices. Finally, total volume of A1 was estimated according to the Cavalieri method, which uses the sum of the areas of systematic, random parallel sections through an object (in this case A1) to calculate an estimate the volume of the object (Gundersen and Jensen, 1987).

It is impossible to generate uniformly random sections consistently orthogonal to the pial surface to ensure clear definition of cortical layers using the SURS approach above. Thus, slabs interleaved with those used to generate mapping sections were selected, and the boundaries of A1 were extended from the mapping sections into adjacent, uncut slabs of tissue. A1 was dissected out of these slabs using cuts perpendicular to the pial surface, and further separated into smaller blocks. Blocks containing A1 from the most rostral, and caudal slabs were each assigned a weight of 1/3, whereas A1 blocks not at the rostral or caudal ends were each assigned a weight of 1. 1/3 was chosen because the boundary of A1 has a positive curvature that is best fit by a cone or pyramid, which is roughly 1/3 the volume of an A1 block not located at a rostral or caudal boundary (Dorph-Petersen et al., 2009), as such blocks are prismoids. Block weights were used during subsequent determination of axon bouton number. SURS was employed to select blocks for additional processing. Each selected block was processed using the Fixed Axis Vertical Rotator (FAVeR) approach, with the central axis of rotation oriented orthogonal to the pial surface, to generate a FAVeR section from the center of the block (Dorph-Petersen and Gundersen, 2003). FAVeR sections were next processed for Nissl substance. Volume of each cortical layer in A1 was derived by multiplying the estimate of the volume fraction of each cortical layer obtained from the FAVeR section with the A1 total volume estimate.

Tissue sections were processed using fluorescence immunohistochemistry for confocal imaging. Vesicular glutamate transporter 1 (VGluT1) protein is present in boutons of cortico-cortical neurons and VGluT2 in boutons of thalamocortical neurons (Fremeau et al., 2001, 2004a,b; Hackett et al., 2011). Thus, anti-VGluT1 (guinea pig, AB5905, Millipore, Billerica, MA, USA) and anti-VGluT2 (rabbit, V2514, HY-19, Sigma-Aldrich, St. Louis, MO, USA) antibodies were used as markers of cortico-cortical and thalamocortical axon boutons, respectively. As expected, VGluT1-IR and VGluT2-IR did not colocalize, whereas VGluT1-IR and VGluT2-IR objects significantly overlapped with the axon bouton marker synaptophysin (Synaptophysin-IR). Neither VGluT1-IR nor VGluT2-IR colocalized with fluorescent objects labeling GAD65-containing inhibitory boutons.

Images were captured with a 1.42 NA 60× oil objective on an Olympus BX51 upright microscope with a DSU spinning disk confocal (Olympus, Center Valley, PA, USA) and collected with SlideBook software (Intelligent Imaging Innovations, Denver, CO, USA). Nevertheless, even with this approach, the resolution was below that necessary to fully separate individual axon boutons, especially in the z-axis of the imaging stack. Therefore, prior to quantification, image stacks were deconvolved. Image stacks were then processed using an iterative masking approach that coupled intensity segmentation with morphological selection (Fish et al., 2008). VGluT1- and VGluT2-IR objects were counted using the associated point rule (Baddeley and Jensen, 2004), with the centroid of each bouton mask serving as the associated point allowing selection of those boutons within an optical dissector.

##### Results

Neither VGluT1-IR nor VGluT2-IR density (Cohort 1 and Cohort 2) nor number (Cohort 2) were altered in deep layer III of A1 in schizophrenia. Similarly, mean VGluT1-IR and VGluT2-IR fluorescence intensity were unchanged in both cohorts. This latter finding indicates not only that bouton VGluT protein levels are unchanged in schizophrenia, but also that the lack of difference in bouton numbers between groups was not due to differences in bouton detectability (Moyer et al., 2013).

#### Study of Glutamate Decarboxylase 65-Immunoreactive Boutons (Moyer et al., 2012)

##### Stereologic methods

Brain specimen acquisition, cutting, sampling and imaging were performed as described in “Study of Vesicular Glutamate Transporter-Immunoreactive Boutons (Moyer et al., 2013)” section, above, in the same two cohorts of cases. As in “Study of Vesicular Glutamate Transporter-Immunoreactive Boutons (Moyer et al., 2013)” section, cases were selected using a combined immunohistochemical-confocal imaging approach. Glutamate decarboxylase 65 (GAD65) synthesizes GABA in axon boutons in response to demand for GABA and during sustained neuronal activity (Kaufman et al., 1991; Esclapez et al., 1994; Tian et al., 1999). A mouse anti-GAD65 antibody (MAB351, Millipore, Billerica, MA, USA) was used to visualize and quantify GAD65 levels in boutons. Image stacks were acquired and processed, and boutons selected for quantification also as described in “Study of Vesicular Glutamate Transporter-Immunoreactive Boutons (Moyer et al., 2013)” section.

##### Results

**Figure 3:**
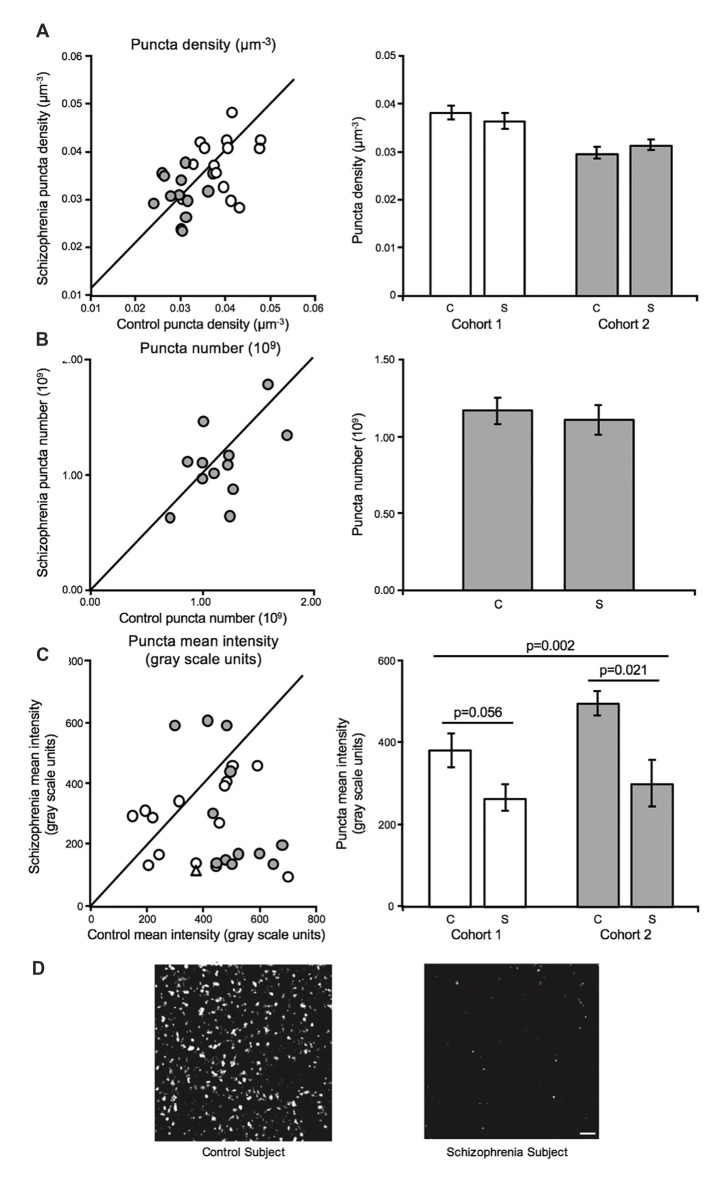
Inhibitory boutons in deep layer III in A1 in schizophrenia (Moyer et al., 2012). For scatterplots: Cohort 1 is denoted by open circles, Cohort 2 is denoted by closed gray circles, and the line represents schizophrenia = unaffected comparison subject values. For bar graphs: C denotes unaffected comparison group, S denotes schizophrenia, and error bars are ± SEM. **(A)** Assessment of biased (Cohort 1) and unbiased (Cohort 2) cohorts revealed putative inhibitory bouton density in deep layer III in A1 is unchanged in schizophrenia. **(B)** Assessment of an unbiased cohort of schizophrenia and unaffected comparison subjects (Cohort 2, closed gray circles) revealed that inhibitory bouton number was also not altered in A1 in schizophrenia. **(C)** Mean intensity of Glutamate decarboxylase 65 immuno-reactive (GAD65-IR) objects was significantly reduced in deep layer III in A1 in schizophrenia in both cohorts. Open triangle in the scatterplot denotes the Cohort 1 subject pair included in the representative micrographs of reduced GAD65-IR in schizophrenia in A1 in **(D)**. Scale bar = 10 μm.

GAD65 immuno-reactive (GAD65-IR) bouton density (Cohort 1 and Cohort 2) and number (Cohort 2) were unaltered in schizophrenia (Figures 3A,B). In contrast, mean bouton GAD65 fluorescence intensity was reduced 40.5% in deep layer III in A1 in schizophrenia (Figures 3C,D; Moyer et al., 2012).

#### Summary of Axon Bouton Studies

In summary, layer III bouton density was significantly reduced in A1 in schizophrenia based on SYP-IR (Sweet et al., 2007). Synaptophysin is found in approximately 95% of cortical boutons (Jahn et al., 1985; Navone et al., 1986) and does not distinguish between excitatory and inhibitory axon boutons (Navone et al., 1986), thus the use of more descriptive markers for boutons were used in subsequent studies. Excitatory bouton density did not appear to be altered in A1 in schizophrenia based on assessments of VGluT1- and VGluT2-IR in two independent cohorts of cases (Moyer et al., 2013). Further, GAD65-IR bouton density was also not altered in two independent cohorts, but GAD65-IR fluorescent intensity was significantly reduced, suggesting that within-bouton GAD65 levels are reduced in A1 in schizophrenia (Moyer et al., 2012). Lower GAD65 levels could indicate decreased GABA synthesis, and thereby contribute to altered fast GABAergic inhibition in auditory circuits which is thought to underlie impaired aSSR entrainment in schizophrenia (Kwon et al., 1999; Light et al., 2006; Krishnan et al., 2009). Future studies are needed to directly test this hypothesis.

Several other bouton types that are SYP-IR have not yet been specifically evaluated in A1 in schizophrenia. These include boutons of chandelier cell inhibitory neurons, sub-populations of somatostatin-expressing and vasoactive intestinal peptide-expressing interneurons that do not express GAD65 (Fish et al., 2011; Rocco et al., 2015), and non-glutamatergic/non-GABAergic boutons (e.g., cholinergic, serotonergic, or dopaminergic boutons). Reduced density of these boutons could contribute to the reduced density of layer III putative axon boutons in schizophrenia, revealed by the assessment of SYP-IR. Finally, published results indicate that synaptophysin itself may be reduced in schizophrenia (Glantz and Lewis, 1997). Thus, it is conceivable that the reduction in SYP-IR bouton density observed in the original article assessing boutons in A1 in schizophrenia (Sweet et al., 2007) could represent reduced detectability of these structures due to lower protein levels in auditory cortex in schizophrenia.

### Studies of Dendritic Spines

#### Study of Spine Quantification Based on Spinophilin Labeling (Sweet et al., 2009)

##### Stereologic methods

A1 and A2 sections adjacent to those previously studied (Cohort 1 as described above in Sections “Study of Synaptophysin-Immunoreactive Boutons (Sweet et al., 2007)”, “Study of Vesicular Glutamate Transporter-Immunoreactive Boutons (Moyer et al., 2013)”, “Study of Glutamate Decarboxylase 65-Immunoreactive Boutons (Moyer et al., 2012)”; Sweet et al., 2007) were selected for processing and assessment of dendritic spine density. Tissue sampling and imaging were performed as described in section “Study of Synaptophysin-Immunoreactive Boutons (Sweet et al., 2007)”, with a few exceptions. Spinophilin is a protein phosphatase 1-binding protein found in dendritic spines (Allen et al., 1997; Muly et al., 2004). A selective affinity-purified polyclonal rabbit antibody raised against a peptide from rat spinophilin (Chemicon, Temecula, CA, USA, AB5669; Amateau and McCarthy, 2002) was used to visualize dendritic spines in deep layer III of A1 and A2 in schizophrenia and unaffected comparison subjects. Evaluation of antibody penetration revealed that this antibody labeled the tissue uniformly, except at each tissue surface (Dorph-Petersen et al., 2001). Spinophilin-immunoreactive (SP-IR) objects were manually counted after capture using a 1.4 numerical aperture 100× oil immersion objective and an optical dissector with x and y dimensions of 3.5 μm and a z height of 50% of the final tissue thickness and guard zones of 25% of the final tissue thickness at each sampling site.

##### Results

The density of SP-IR puncta was reduced by 27.2% in deep layer III of A1 in schizophrenia. Similarly, SP-IR puncta density was also reduced by 22.2% in deep layer III of A2 in schizophrenia. Further, SP-IR density was correlated with density of synaptophysin-immunoreactive axon boutons (Sweet et al., 2009).

These findings were consistent with previous results reporting reduced dendritic spine density in regions of neocortex and of the hippocampal formation implicated in schizophrenia (Moyer et al., 2015). As described in section “Study of Synaptophysin-Immunoreactive Boutons (Sweet et al., 2007)”, because of the *in vivo* imaging evidence of reduced gray matter volume of auditory cortex in schizophrenia, the reduced density of dendritic spines was interpreted to reflect a reduction in spine number, despite the fact that A1 volume was not estimated in these subjects. Previous studies using Golgi methods revealed reduced numbers of dendritic spines per dendritic length in PFC (Glantz and Lewis, 2000) and in another region of temporal cortex, Brodmann area 38, in schizophrenia (Garey et al., 1998). Similar to other regions, dendritic spine number was predicted to be decreased in A1 in this illness. Reduced number of dendritic spines in layer III of A1 in schizophrenia could reflect fewer spines per dendrite, reduced total dendritic length of pyramidal cells, reduced numbers of pyramidal cells, or their combination.

A further comment is warranted regarding the potentially conflicting findings of: (1) correlated reductions in dendritic spine density and in density of SYP-IR boutons; and (2) no reduction in excitatory (VGluT1- and VGluT2-IR) boutons. Dendritic spine density is shaped by activity-dependent glutamate signaling (Nägerl et al., 2004; Zhou et al., 2004; Xie et al., 2005). Spine density has been shown to be reduced when glutamatergic signaling is disrupted (Matthews et al., 1976; Cheng et al., 1997; McKinney et al., 1999). Because reduced synaptophysin protein levels can impair glutamate signaling (Kwon and Chapman, 2011), it is plausible that synaptophysin levels are reduced within VGluT1- or VGluT2-positive boutons. Therefore, reduced synaptophysin may contribute to dendritic spine reductions in auditory cortex in schizophrenia. In a future study, this hypothesis could be readily tested using a multi-label immunohistochemistry design with anti-VGluT1, -VGluT2, and -synaptophysin antibodies, in combination with SURS sampling and quantitative fluorescence microscopy.

#### Study of Spine Quantification Based on Spinophilin-Phalloidin Double Labeling (Shelton et al., 2015)

##### Stereologic methods

Two cohorts, each comprised of one schizophrenia and one unaffected comparison group, were assessed. The first cohort consisted of the same subjects, and used the same methods of tissue sampling as described for Cohort 2 in sections “Study of Vesicular Glutamate Transporter-Immunoreactive Boutons (Moyer et al., 2013)” and “Study of Glutamate Decarboxylase 65-Immunoreactive Boutons (Moyer et al., 2012)” above. Cases in the second cohort were sampled using a simpler SURS approach. In brief, the left STG was dissected, reassembled in low-melt agarose, and cut into 3 mm slabs orientated orthogonal to Heschl’s gyrus. Every other slab with a random start was selected and A1 mapped using adjacent sections stained for Nissl substance, AChE, and parvalbumin. The sections adjacent to the mapping sections were selected for assay SURS. A1 tissue sections were incubated in an anti-spinophilin antibody (polyclonal, AB5669; EMD Millipore, Billerica, MA, USA), to visualize spine heads (Allen et al., 1997; Muly et al., 2004). Additionally, the tissue sections were incubated in phalloidin conjugated to AlexaFluor568 (A12380; Invitrogen Corp., Carlsbad, CA, USA). Phalloidin is a mushroom toxin that binds filamentous actin which is enriched in dendritic spines (Capani et al., 2001). Colocalized SP-IR and phalloidin-labeled fluorescent objects closely resemble dendritic spines in human postmortem A1 tissue (Shelton et al., 2015). A spinophilin + phalloidin dual-labeling approach was used in the current study to identify and exclude potential off-target phalloidin-labeled and SP-IR objects. Two labels were used to detect putative spines because each label separately emits non-spine fluorescence. For example, phalloidin-labeling is sometimes observed in dendrites (presumably labeling filopodia outgrowths; Willig et al., 2014). Images were captured using a 1.42 numerical aperture 60× oil supercorrected objective on an Olympus BX51 upright microscope with a DSU spinning disk confocal (Olympus, Center Valley, PA, USA) with stage x- and y-axis movement controlled by Stereo Investigator (Microbrightfield Inc., Natick, MA, USA). Image stacks were collected using SlideBook software (Intelligent Imaging Innovations, Denver, CO, USA) which controlled z-axis movement. After collection, images of fluorescently labeled objects were processed as described in sections “Study of Vesicular Glutamate Transporter-Immunoreactive Boutons (Moyer et al., 2013)” and “Study of Glutamate Decarboxylase 65-Immunoreactive Boutons (Moyer et al., 2012)” with one addition. After deconvolution, object edges were sharpened by taking the difference between images convolved at different 2 SD of the Gaussian distribution (σ1 = 0.7; σ2 = 2.0; Kirkwood et al., 2013), after which point the images underwent iterative masking via intensity segmentation with morphological selection. Dendritic spines were defined as phalloidin masked objects overlapping by one or more voxels of a spinophilin masked object. Spines were counted if the centroid of the phalloidin masked object fell inside the dissector, consistent with the associated point rule (Baddeley and Jensen, 2004).

##### Results

**Figure 4:**
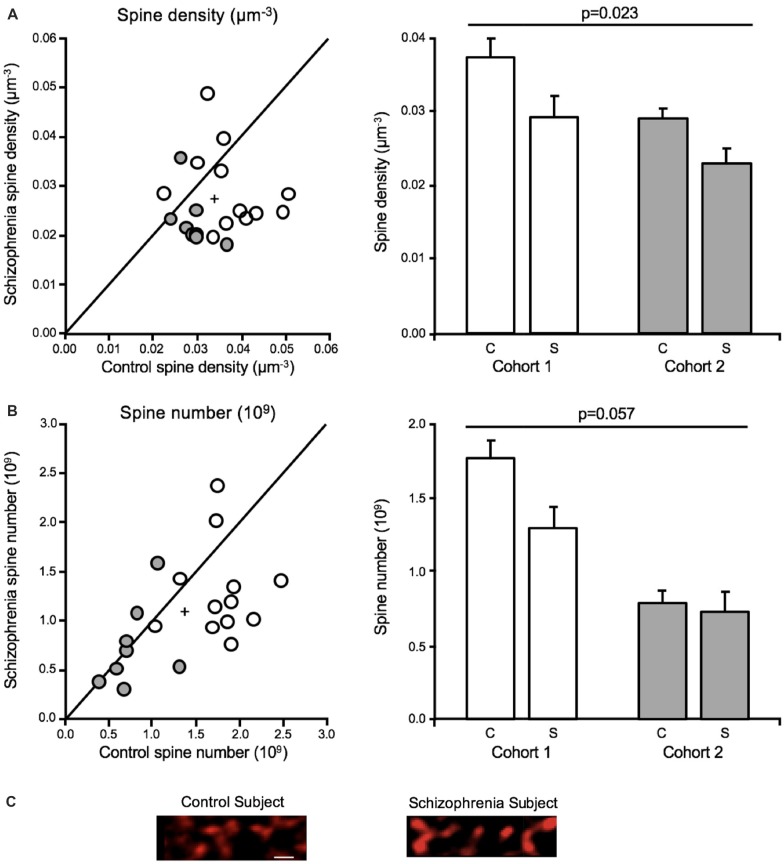
Dendritic spine alterations in deep layer III in A1 in schizophrenia (Shelton et al., 2015; MacDonald et al., 2017). For scatterplots: Cohort 1 is denoted by open circles, Cohort 2 is denoted by closed gray circles, the group mean is indicated by the cross, and the line represents schizophrenia = unaffected comparison subject values. For bar graphs: C denotes unaffected comparison group, S denotes schizophrenia, and error bars are ± SE. **(A)** Assessment of cohorts 1 and 2 revealed mean dendritic spine density was reduced in deep layer III in A1 in schizophrenia. **(B)** Mean spine number was also found to be significantly reduced in deep layer III in A1 in schizophrenia. **(C)** Representative micrographs of phalloidin-labeled objects from one pair comprising one unaffected comparison case and one schizophrenia case revealing putative dendritic spine loss in A1 in schizophrenia. Scale bar = 1 μm.

Both dendritic spine density and number were significantly reduced in deep layer III in A1 in schizophrenia (Figures 4A–C), consistent with the earlier report that found decreased spine density in this region in schizophrenia (Sweet et al., 2009). The earlier report found a reduction in SP-IR density of 27.2%, whereas the current study reported a 19.2% reduction in mean spine density in deep layer III in A1 in schizophrenia. Off-target labeling of the anti-spinophilin antibody could explain the discrepancy in the magnitude of spine density reductions observed across studies. Significantly, the current study provided for the estimation of dendritic spine number in addition to density (Shelton et al., 2015). Thus, this study showed, for the first time in any brain region, that dendritic spine number is reduced in schizophrenia.

#### Study of Pyramidal Cell Number (Dorph-Petersen et al., 2009)

##### Stereologic methods

The current study was conducted using the Cohort 2 subjects described above in sections “Study of Vesicular Glutamate Transporter-Immunoreactive Boutons (Moyer et al., 2013)” and “Study of Glutamate Decarboxylase 65-Immunoreactive Boutons (Moyer et al., 2012)” and detailed in the *Stereological methods* portion of section “Study of Vesicular Glutamate Transporter-Immunoreactive Boutons (Moyer et al., 2013)”, above, with a minor adjustment. A FAVeR (weighted) sampling scheme was used to estimate pyramidal cell number in layer III in A1. Pyramidal cells were identified in the FAVeR sections stained for Nissl substance, and counted using an optical dissector in Stereo Investigator (Microbrightfield; Gundersen, 1986).

Volume by cortical layer in A1 and total A1 volume were estimated for postmortem schizophrenia and unaffected comparison cases. With total reference volume estimations, the authors of the current study were able to estimate pyramidal cell number in A1 in schizophrenia, for the first time.

##### Results

**Figure 5:**
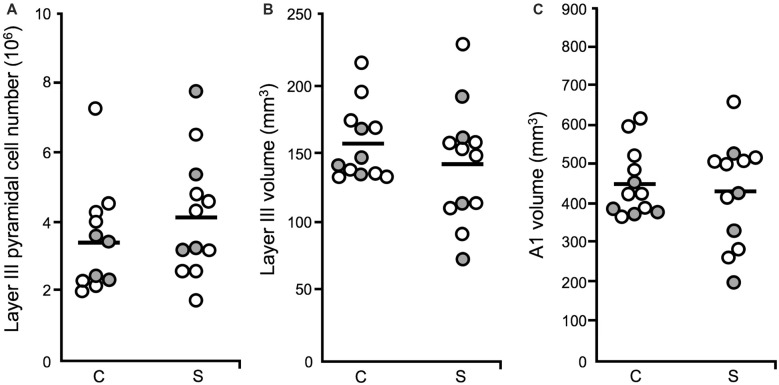
Layer III pyramidal cell number, layer III volume and total A1 volume (Dorph-Petersen et al., 2009). For all plots: diagnostic subject pairs with one member diagnosed with schizophrenia are denoted by open circles and subject pairs with one member diagnosed with schizoaffective disorder are denoted by closed gray circles. **(A)** The number of pyramidal cells in layer III in A1 was unchanged in schizophrenia.** (B)** The volume of layer III in A1 in schizophrenia was not significantly different across diagnostic groups. **(C)** Total A1 volume did not significantly differ in schizophrenia relative to unaffected comparison cases.

A1 layer III pyramidal cell density was increased in schizophrenia, relative to unaffected comparison cases. In contrast, pyramidal cell number did not differ significantly across diagnostic groups (Figure 5A), in agreement with previous reports that found no change in neuron number in cerebral cortex (Pakkenberg, 1993), PFC (Thune et al., 2001) nor anterior cingulate (Stark et al., 2004) in schizophrenia. Neither layer III A1 volume (Figure 5B), nor total A1 volume were found to be significantly reduced in schizophrenia (Figure 5C). Increased pyramidal cell density, with no change in pyramidal cell number, was interpreted to mean that STG gray matter loss in schizophrenia is due to the loss of neuropil components such as dendritic spines, rather than to the loss of the number of pyramidal cells.

## Discussion

**Figure 6:**
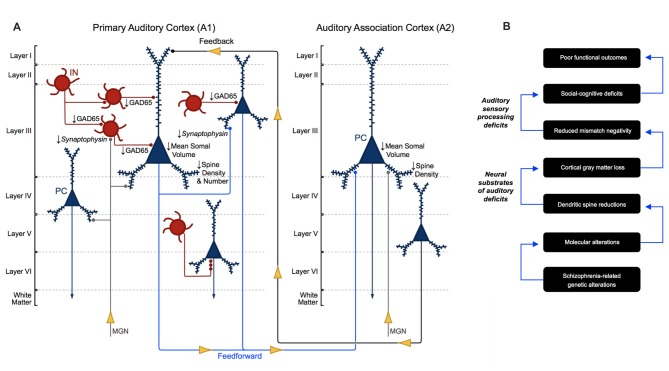
Morphometric alterations in auditory cortex in schizophrenia. **(A)** Neuron pathology in schizophrenia as revealed by human postmortem studies of auditory cortex. Mean somal volume of PCs in deep layer III in A1 and A2 in schizophrenia were found to be significantly reduced. Dendritic spine density was reduced in deep layer III of A1 and A2, reflecting reduced number of dendritic spines in deep layer III of A1 in schizophrenia. GAD65 levels were reduced in deep layer III boutons in A1 in schizophrenia, although the specific interneuron cell types affected are not currently known. **(B)** Proposed model for auditory cortex related dysfunction in schizophrenia, linking genetic risk to anatomical alterations, auditory sensory processing deficits and poor functional outcomes.

Auditory sensory processing deficits correlate with gray matter loss in Heschl’s gyrus in schizophrenia (Salisbury et al., 2007) and are now understood to reflect structural alterations, in particular, altered neuron morphology in A1 in schizophrenia (Leitman et al., 2007, 2010; Petkova et al., 2014; Javitt and Sweet, 2015). To date, human postmortem studies have revealed several important neuronal morphological aberrations in auditory cortex in schizophrenia (Figure 6A). Pyramidal cells in A1 and A2 deep layer III exhibit reduced somal volume (Sweet et al., 2003, 2004). Findings regarding axon boutons in deep layer III in auditory cortex in schizophrenia are mixed. An earlier report found reduced putative bouton density (Sweet et al., 2007), while later publications reported no change in the density of excitatory and inhibitory boutons (Moyer et al., 2012, 2013). Consistent with the reduced neuropil hypothesis of schizophrenia (Selemon and Goldman-Rakic, 1999; Glausier and Lewis, 2013), the number and density of dendritic spines were found to be reduced in deep layer III of A1 in schizophrenia in three independent cohorts of subjects (Sweet et al., 2009; Shelton et al., 2015).

Unlike in other diseases, such as Alzheimer’s disease (Penzes et al., 2011), in A1 in schizophrenia dendritic spine loss is not accompanied by neuron loss. Specifically, layer III pyramidal cell number was confirmed to be unaltered in A1 in this illness (Dorph-Petersen et al., 2009), indicating that spine loss in this region likely reflects fewer spines per pyramidal cell. Finally, gray matter loss in auditory regions in schizophrenia has been shown to progressively worsen over time in subjects with schizophrenia, raising concerns that progressive gray matter loss is a consequence of damage to cellular structures from long-term antipsychotic use (Vita et al., 2012). Importantly, postmortem studies of long-term haloperidol exposed non-human primates have found no significant evidence that the morphology of auditory cortical neurons change as a function of antipsychotic exposure, indicating that gray matter alterations in auditory cortex in schizophrenia are not simply a result of effects of long-term antipsychotic use on these structures (Sweet et al., 2007; Moyer et al., 2012, 2013; Shelton et al., 2015).

Unique among studies of auditory-related deficits in schizophrenia, postmortem studies provide the resolution needed to identify anatomical alterations at the neuron level. The studies detailed in this review revealed several important morphometric alterations in auditory cortex in schizophrenia, and in doing so advanced the field. However, two limitations of these studies must be noted. First, all of the studies detailed in this review examined auditory cortical tissue from the left hemisphere, only (Sweet et al., 2003, 2004, 2007, 2009; Dorph-Petersen et al., 2009; Moyer et al., 2012, 2013; Shelton et al., 2015) whereas the basic auditory processing deficits measured using *in vivo* methods from human subjects with schizophrenia are bilateral (Leitman et al., 2010). Follow up studies are needed to assess neuropathology in auditory cortex in schizophrenia in the right hemisphere. Second, these data suggest as a whole that the function of layer III pyramidal cells are altered in auditory cortex in schizophrenia. However, the immunolabeling strategies used, for example, in the dendritic spine studies could not indicate the laminar location of the cell bodies that corresponded to the altered spines (Sweet et al., 2009; Shelton et al., 2015). Therefore, although most dendritic spines in layer III arise from layer III pyramidal cells, reduction of dendritic spine on auditory cortex layer III pyramidal cells in schizophrenia has not been specifically demonstrated.

Incorporating stereological methods into postmortem studies has been instrumental in revealing neuronal morphological aberrations in auditory cortex in schizophrenia. These methods have enabled our group to utilize unbiased sampling of tissue as well as the unbiased estimation of the structural features of neurons, including axon bouton density and number, dendritic spine density and number, and pyramidal cell number. Likewise, rigorous inclusion of stereological techniques has made it possible for our group (Dorph-Petersen et al., 2009) and others (Smiley et al., 2013) to accurately estimate individual cortical layer volumes within A1 and A1 total volume. In sum, stereological methods have been critical for the collection and publication of a body of work about neuronal alterations in auditory cortex in schizophrenia that provide a strong foundation upon which future studies will rely.

One such study is a recent human postmortem study of dendritic spines in A1 in schizophrenia, which performed a secondary analysis of the data described in section “Study of Spine Quantification Based on Spinophilin-Phalloidin Double Labeling (Shelton et al., 2015)” which revealed that reduced dendritic spine density in A1 in schizophrenia is restricted to the smallest dendritic spines (MacDonald et al., 2017). *In vivo* imaging of dendritic spine dynamics in animal models indicate that small spines are largely new and/or transient, whereas large spine are typically mature/persistent (Holtmaat et al., 2005). Selective reduction of nascent dendritic spines could indicate that altered spine morphology in schizophrenia is due to aberrant spinogenesis or spine stabilization, rather than, as was historically assumed, over-pruning of mature spines during adolescent development (Boksa, 2012).

Reduced dendritic spine density may function as an intermediate, anatomical phenotype for schizophrenia, a key component in a hierarchical model that could link genetics, at one extreme, to functional outcomes, at the other (Figure 6B). Many schizophrenia risk genes encode synaptic proteins and those involved in excitatory (Ca^2+^) signaling in dendrites (Purcell et al., 2014; Schizophrenia Working Group of the Psychiatric Genomics Consortium, 2014; Heyes et al., 2015). Thus, one promising future direction for the assessment of A1 cellular pathology in schizophrenia involves investigating potential mechanisms associated with known genetic risk for schizophrenia, that could lead to reduced small dendritic spine density, as is observed in A1 in this disease. Our group recently showed that levels of a peptide shared among voltage-gated calcium channel (VGCC) β subunits (Ca_V_β) was inversely correlated with the density of small spines. Overexpressing *CACNB4*, the gene that encodes Ca_V_β4, which is present in the temporal cortex, and is a critical regulator of VGCC activity (Dolphin, 2012), in primary neuronal culture resulted in reduced density of small, but not large, dendritic spines (MacDonald et al., 2017). Future studies should observe the effects of overexpressing *CACNB4* in a model organism during adolescence, a period that is associated with schizophrenia onset as well as system-wide spine number changes in normative development (Penzes et al., 2011). The functional outcomes of individuals with schizophrenia have remained largely unchanged since the introduction of antipsychotics, likely due in part to the fact that we do not fully understand the pathophysiology of this illness (Insel, 2010). Probing relationships between Ca_V_β4 (and other proteins like it) and dendritic spine morphology has the potential to elucidate an important, anatomical phenotype of schizophrenia, which may be a final common pathway for auditory impairment in schizophrenia. Finally, examining such relationships could lead to the identification of drug targets or provide other critical information for the development of superior strategies to treat or prevent auditory sensory processing deficits in schizophrenia.

## Author Contributions

All studies reviewed took place in the lab of RAS. EMP did not contribute to any of the studies reviewed (hence, lack of authorship on the published works). EMP drafted the current review with significant direction from and revision by RAS. EMP and RAS approve the version to be published. The content is solely the responsibility of the authors and does not necessarily represent the official views of the National Institute of Mental Health, the National Institutes of Health, the Department of Veterans Affairs, or the United States Government.

## Conflict of Interest Statement

The authors declare that the research was conducted in the absence of any commercial or financial relationships that could be construed as a potential conflict of interest.
